# Chain architectures of various cellulose-based antiscalants on the inhibition of calcium carbonate scale

**DOI:** 10.1038/s41598-020-78408-w

**Published:** 2020-12-14

**Authors:** Wei Yu, Hu Yang

**Affiliations:** State Key Laboratory of Pollution Control and Resource Reuse, School of the Environment, Nanjing University, Nanjing, 210023 People’s Republic of China

**Keywords:** Environmental sciences, Environmental chemistry

## Abstract

Two series of cellulose-based antiscalants with different chain architectures, i.e., linear carboxymethyl cellulose (CMC) and branch-shaped carboxymethyl cellulose-*graft*-poly(acrylic acid) (CMC-*g*-PAA), were synthesized. The carboxyl groups were distributed on CMC backbone but mainly on the grafted chains of CMC-*g*-PAA. The addition of CMC and CMC-*g*-PAA can both increase the surface energy of CaCO_3_ scale and decrease its crystal nucleation rate, thereby inhibiting CaCO_3_ scale formation. The structural effects of these cellulose-based antiscalants, especially the chain architectures, on the scale inhibition were investigated in detail. High degree of carboxymethyl substitution caused better inhibition effect of linear CMC. However, CMC-*g*-PAA with an appropriate content of carboxyl groups but high average number of PAA grafted chains can achieve high inhibition performance. Besides, with similar contents of carboxyl groups, CMC-*g*-PAA showed much better inhibition performance than CMC due to the distinct multi-dimensional spatial structure of graft copolymer in solution, causing the enhanced chelation and dispersion effects. Characterization of CaCO_3_ crystal by scanning electron microscopy and X-ray diffraction confirmed that crystal distortion effect obviously existed in CMC but quite minor in CMC-*g*-PAA. The differences between the scale-inhibition performance of CMC and CMC-*g*-PAA should be attributed to the different scale-inhibition mechanisms originated in their distinct chain architectures.

## Introduction

Effective utilization of water resources is a concern owing to increased demand of fresh water from rapid economic development^1,2^. Currently, desalination of seawater or brackish water is one of important technologies, increasing amounts of fresh water and realizing the utilization of water resources^1,3,4^. Scale formation is unavoidable when solute concentrations are enhanced to a certain extent, exerting detrimental effects on desalination processes such as equipment damage, operation shutdown and frequent cleaning^3,5–8^. Common inorganic scales mainly include calcium carbonate, magnesium carbonate, calcium sulfate and silica-based scale^9–12^. Calcium carbonate scale can easily form and attach to equipment surface as dense deposits, due to its ubiquity in various types of feed water and its low solubility^5,6,11,13,14^. The solubility of calcium carbonate depends on the pH, which decreases continuously from low to high pH, as shown in the following equation: Ca^2+^  + HCO_3_^−^ ↔ H^+^  + CaCO_3_^3,15,16^. Precipitation and softening by adding alkaline substances or acidification can be applied to mitigated calcium carbonate scaling^5^. However, wastes disposal from softening and aggravated corrosion caused by acidification cannot be ignored^2,5,17^.

Antiscalant can effectively inhibit scale formation based on chelation, dispersion, crystal distortion and threshold effects, thus widely applied in scaling control^3,5,18^. Traditional commercial antiscalants mainly refer to phosphorus-containing antiscalants, which, however, should be restricted due to phosphorus emission causing adverse environmental impacts^16,19,20^. Phosphorus-free antiscalants, especially natural polymer-based antiscalants, have attract much attention in recent years^16,20,21^. In addition to polyaspartic acid^16,21–23^, polysaccharide-based antiscalants, as an important class of natural polymer-based antiscalants, showed notably potential application prospect due to their advantages of low cost, good biodegradability and environmental-friendliness^16,21^. However, some polysaccharides themselves generally have poor inhibition performance owing to lack of effective functional groups and poor solubility^24–26^. Appropriate chemical modification on these polysaccharides can be applied to introduce available functional groups and obtain suitable molecular structures, thus significantly enhancing their inhibition performance^24^. Etherification and graft polymerization are two common chemical modification methods to obtain natural polymers derivatives with specific properties^27–30^. Some polysaccharide derivatives obtained from etherification and graft polymerization, such as carboxymethyl cellulose (CMC), carboxymethyl starch, carboxymethyl inulin, and starch-*graft*-poly(acrylic acid), have been used as effective antiscalants and exhibited improved antiscaling performance^24,25,31–33^.

Different structural morphologies of antiscalants can be obtained using various modification methods, which would lead to distinct material properties and determine their final inhibition performance^24,25,31,33–36^. The relationship between molecular structure and scale-inhibition performance should be well understood to guide the design and fabrication of high-performance antiscalants, especially for polymeric antiscalants with multiplicity of structural features^25,37–41^. The types and contents of selected functional groups on the polymeric antiscalants have evident influences on their scale-inhibition performance^24,25,33,41^; besides, different chain architectures of polymers, including linear, branching, and star-like structures, also have notable effects on their application performance^37,42^. The distributions of the same functional groups on the polymers, with the similar contents but at different chain sites such as at the backbone or at the branched chains, exhibit different chain architectures and usually result in varied application performance^37,42^. However, little work has focused on the effects of chain architectures of the polymeric antiscalants on the scale-inhibition performance until now.

In this work, two series of cellulose-based antiscalants all containing carboxyl groups but distributed at different chain sites have been synthesized, i.e. linear CMC with carboxyl groups on cellulose backbones and carboxymethyl cellulose-*graft*-poly(acrylic acid) (CMC-*g*-PAA) with carboxyl groups mainly distributed on branched chains, which exhibited far different chain architectures. The scale-inhibition performance of aforementioned various cellulose-based antiscalants against CaCO_3_ scale have been investigated and compared in detail in terms of their structural characteristics. In addition to the contents of carboxyl groups, i.e., the degree of carboxyl substitution of CMC and grafting ratio of CMC-*g*-PAA, the effects of their chain architectures, i.e., the distinct distribution of the carboxyl groups on the linear and branched chains, have been studied. Crystal morphologies of CaCO_3_ deposits were characterized by scanning electron microscopy (SEM) and X-ray diffraction (XRD). The induction time (*t*_ind_) of CaCO_3_ crystal growth was measured by monitoring pH change in the scaling process. Combination of the apparent inhibition performance and aforementioned characterizations, the inhibition mechanisms of those cellulose-based antiscalants associated with their distinct molecular structures have been discussed in detail.

## Results and discussion

### Structure characteristics of various cellulose-based antiscalants

**Figure 1:**
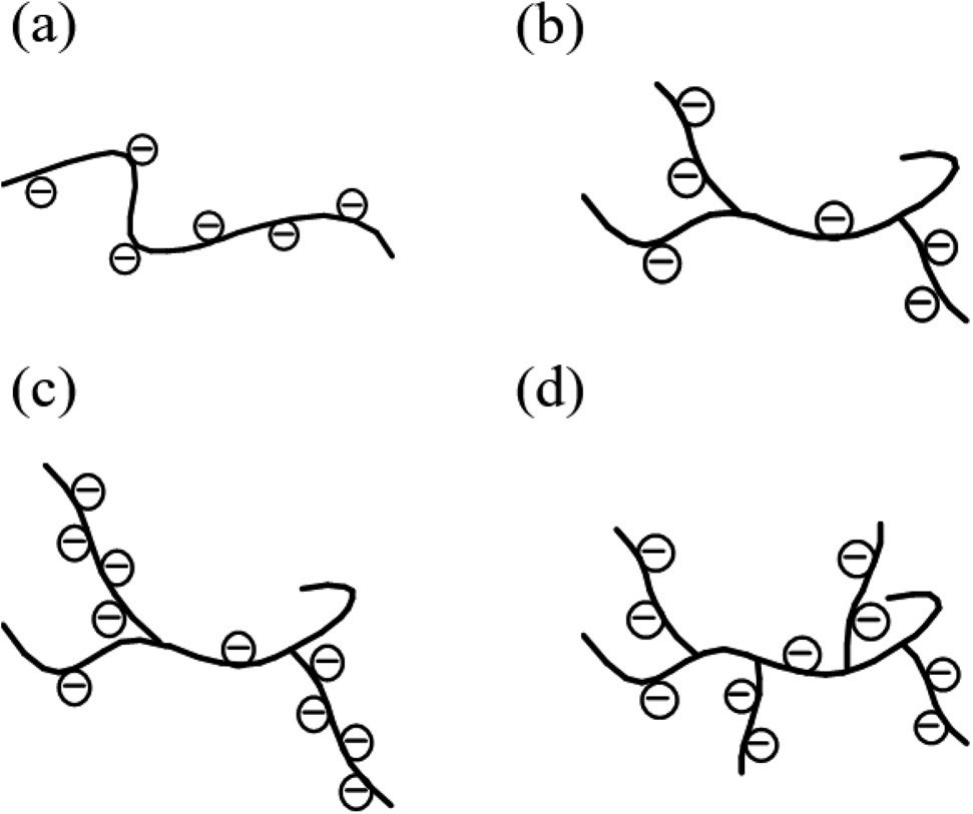
The structural morphologies of (**a**) CMC and (**b**–**d**) different CMC-*g*-PAA samples, (**b**, **c**) CMC-*g*-PAA with similar numbers of grafted chain but different grafting ratio: (**b**) low and (**c**) high grafting ratio; and (**c**, **d**) CMC-*g*-PAA with the similar grafting ratio but different grafted-chain distributions: (**c**) longer but fewer and (**d**) shorter but more grafted chains.

Two series of cellulose-based antiscalants with different chain architectures, CMC and CMC-*g*-PAA, were synthesized by etherification and graft polymerization, respectively^24,27,43^, which are described in detail in Supplementary Information S1. According to FTIR and ^1^H NMR characterizations (Supplementary Figs. S1–S2 and Supplementary Information S1), those various cellulose-based antiscalants were successfully obtained. The structural morphologies of CMC and CMC-*g*-PAA were illustrated in Fig. 1. CMC is a linear polymer with carboxyl groups on the backbone^43^, but CMC-*g*-PAA is a graft copolymer and the carboxyl groups contained is mainly distributed on the branched chains^27^. Furthermore, the detailed structural parameters, including the degree of carboxyl substitution of CMC and the grafting ratio and grafted-chain distributions of CMC-*g*-PAA (the average number of grafted chain per graft copolymer, N; and the average number of the grafted monomers per grafted chain, L), were measured and estimated according to Supplementary Information S1, which were listed in Tables 1 and 2. The degrees of carboxymethyl substitution of CMC increased from CMC(1) to CMC(6) (Table 1). As for CMC-*g*-PAAs (Table 2), CMC-*g*-PAA(1)–CMC-*g*-PAA(7) had similar N but increased grafting ratios and L, while CMC-*g*-PAA(a)–CMC-*g*-PAA(d) exhibited similar grafting ratios and carboxyl group contents but different N and L. The degree of carboxyl substitution of CMC and the grafting ratio of CMC-*g*-PAA reflected their contents of carboxyl groups, which were, accordingly, estimated and listed in Tables 1 and 2, respectively, for better comparison. The calculated contents of carboxyl groups were fully proportional to the degree of carboxyl substitution of CMC and the grafting ratio of CMC-*g*-PAA, respectively.

**Table 1:** Summary of the preparation conditions, structural parameters and the scale-inhibition performance of various CMC samples with different degrees of carboxyl substitution against CaCO_3_ scales based on static tests and crystallization process.

Samples	Molar feeding ratio of cellulose to choroacetic acid	Degree of carboxyl substitution	Content of carboxyl group (mmol g^−1^)	Intrinsic viscosity [*η*] (g L^−1^)	Optimal dose (mg L^−1^)	Inhibition efficiency (%)^a^	Induction time (min)^b^
CMC(1)	1:0.25	0.20	1.1986	0.0286	100.0	26.0 ± 2.2	3.33
CMC(2)	1:0.5	0.35	1.7423	0.0347	60.0	45.6 ± 4.2	7.00
CMC(3)	1:1	0.54	2.3149	0.0601	60.0	60.2 ± 3.5	16.00
CMC(4)	1:1.2	0.71	2.9219	0.0680	60.0	81.2 ± 4.2	21.13
CMC(5)	1:2	1.52	4.9382	0.0707	40.0	91.6 ± 4.2	25.33
CMC(6)	1:4	1.90	5.4298	0.0899	20.0	98.3 ± 0.3	29.67

^**a**^Scale-inhibition efficiencies of various CMC samples in static test, at their optimal doses;

^**b**^The induction time was obtained in the presence of 2.0 mg L^−1^ CMC.

**Table 2:** Summary of the preparation conditions, structural parameters and the scale-inhibition performance of various CMC-*g*-PAA samples with different grafting ratios and grafted-chain distributions against CaCO_3_ scales based on static tests and crystallization process.

Samples	Mass feeding ratio of CMC to AA	Initiator (g)	Grafting ratio (%)	Content of carboxyl group (mmol g^−1^)	Intrinsic viscosity [*η*] (g L^−1^)	Average number of grafted-chain (N)^a^	Average length of the grafted-chain (L)	Optimal dose (mg L^−1^)	Inhibition efficiency (%)^b^	Induction time (min)^c^
CMC-*g*-PAA(1)	1:0.15	0.5	24	3.2696	0.0242	6	40	8.0	94.8 ± 3.8	35.00
CMC-*g*-PAA(2)	1:0.3	0.5	59	4.3999	0.0248	6	98	6.0	95.3 ± 3.9	48.00
CMC-*g*-PAA(3)	1:0.5	0.5	118	5.8478	0.0264	6	196	8.0	98.4 ± 0.5	73.00
CMC-*g*-PAA(4)^d^	1:1	0.5	241	7.7918	0.0297	6	402	8.0	93.5 ± 4.0	43.67
CMC-*g*-PAA(5)	1:1.5	0.5	375	9.0596	0.0427	6	625	20.0	97.2 ± 1.7	36.17
CMC-*g*-PAA(6)	1:2	0.5	477	9.7182	0.2060	6	795	40.0	81.6 ± 4.0	4.58
CMC-*g*-PAA(7)	1:3	0.5	793	10.9575	0.2984	6	1321	40.0	78.9 ± 5.0	4.17
CMC-*g*-PAA(a)	1:1	0.1	214	7.4537	0.0372	1	2143	10.0	92.7 ± 1.6	33.00
CMC-*g*-PAA(b)	1:1	0.25	231	7.6609	0.0310	3	768	8.0	92.5 ± 1.9	44.33
CMC-*g*-PAA(c) ^d^	1:1	0.5	241	7.7918	0.0297	6	402	8.0	93.5 ± 4.0	43.67
CMC-*g*-PAA(d)	1:1	0.8	246	7.8552	0.0267	9	274	6.0	93.4 ± 2.2	47.50

^**a**^The average number of grafted-chain per 1000 saccharide rings.

^**b**^Scale-inhibition efficiencies of various CMC-*g*-PAA samples in static test, at their optimal doses.

^**c**^The induction time was obtained in the presence of 1.0 mg L^−1^ CMC-*g*-PAA.

^d^CMC-*g*-PAA(4) and CMC-*g*-PAA(c) are the same sample.

Tables 1 and 2 also present the intrinsic viscosity ([*η*]) of CMCs and CMC-*g*-PAAs. [*η*] presents the hydrodynamic volume of a polymer in solution and can sometimes reflect the molecular weight based on Mark-Houwink-Sakurada equation, i.e., [*η*] = KM_v_^α^^34,44,45^. According to Tables 1 and 2, the [*η*] of CMCs increased from CMC(1) to CMC(6), indicating the molecular weight of CMCs increased with increasing substitution degree because various CMCs exhibit similar structures and compositions and thus have similar two Mark-Houwink-Sakurada constants (K and α). Similarly, CMC-*g*-PAAs with higher grafting ratio had larger [*η*] and higher molecular weight. Nevertheless, the molecular weight of linear CMC and branched CMC-*g*-PAA could not be compared, since their far different molecular structures caused highly different Mark-Houwink-Sakurada constants^34,44^. Compared to CMCs, CMC-*g*-PAA with low grafting ratio showed lower [*η*], besides, CMC-*g*-PAA(d) with more branched PAA chains also showed smaller [*η*] in comparison to CMC-*g*-PAA(a)–CMC-*g*-PAA(c) with similar grafting ratio (Table 2), because branched polymer usually shows compacted chain confirmation in solution, smaller hydrodynamic volume, and lower [*η*] than the corresponding linear polymer with similar molecular weight and composition^34,44,46^.

### Effect of dose on inhibiting CaCO_3_ scale formation

**Figure 2:**
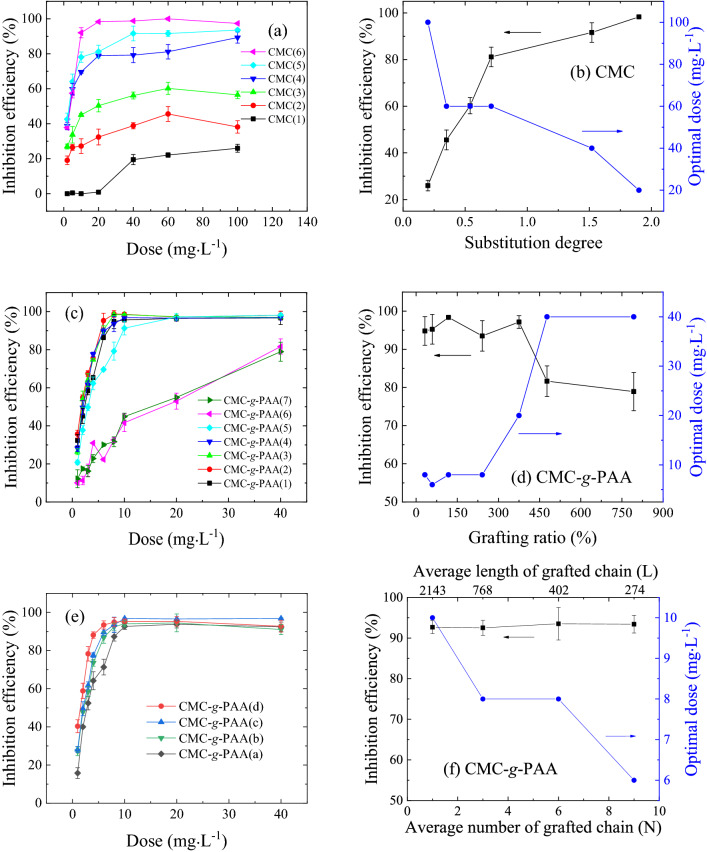
Effects of dose on the scale-inhibition efficiency of (**a**) different CMC and (**c**, **e**) various CMC-*g*-PAA with (**c**) different grafting ratio and (**e**) different grafted-chain distributions against CaCO_3_ scale in static tests. According to (**a**), (**c**) and (**e**), the structural effects, i.e. (**b**) degrees of carboxyl substitution of CMC, (**d**) grafting ratio and (**f**) grafted-chain distribution of CMC-*g*-PAA, on the inhibition efficiency of these cellulose-based antiscalant against CaCO_3_ scale at their optimal doses were summarized.

The dose effects of different cellulosed-based antiscalants on the inhibition of CaCO_3_ scale formation were studied, which were presented in Fig. 2. Based on Fig. 2, the optimal doses and corresponding inhibition efficiencies of CMC and CMC-*g*-PAA samples are all listed in Tables 1 and 2. Accordingly, most of cellulosed-based antiscalants exhibited good inhibition efficiency higher than 80% due to the enhanced chelation and dispersion effects after introducing carboxyl groups onto cellulose as active inhibition sites^4,24,25,33^. The inhibition performance of different cellulose-based antiscalants was strongly dependent on their structural features (Fig. 2 and Tables 1 and 2), which would be discussed in the following sections.

However, the inhibition performance of most cellulosed-based antiscalants exhibited similar dose dependence (Fig. 2). The inhibition efficiency increased with the increase of dose at the beginning, and then the inhibition efficiency did not change or even decreased slightly with further increase of dose after the maximum efficiency was achieved. Further increase of antiscalant dose did not improve or even reduce the inhibition performance, which may be owing to the formation of intermolecular hydrogen bonds and enhanced bridging flocculation effect resulting from excessive antiscalants^24,25,33^. This finding indicated that suitable antiscalant dose was essential for achieving good inhibition performance and cost, which was fully consistent with previous reports^20,25,33,47,48^.

Nevertheless, the inhibition efficiencies of CMC(1), CMC-*g*-PAA(6), and CMC-*g*-PAA(7) were not only relatively low in their respective series but also continuously increasing with the increase of dose (Fig. 2a,c), further indicating their lower inhibition efficiencies. The inefficient inhibition performance of CMC(1) was mainly due to its low content of carboxyl groups (Table 1)^33^; while CMC-*g*-PAA(6) and CMC-*g*-PAA(7) with high grafting ratios and high molecular weights (Table 2) had enhanced bridging flocculation but weakened dispersion effects and resultantly low scale-inhibition performance^25,49^. Thus, the maximum efficiencies of these three cellulose-based antiscalants were not achieved in the measured dose range and more doses were still required (Fig. 2a,c).

### Effect of degree of carboxyl substitution of CMC

Carboxyl group is the main active group to inhibit CaCO_3_ scale formation due to its good chelation effect with scale-forming ions^50,51^. The content of carboxyl groups in antiscalants would thus have notable influences on their inhibition performance. Degree of carboxyl substitution and grafting ratio directly reflect the contents of carboxyl groups in CMC and CMC-*g*-PAA, respectively. The effects of these two structural factors on the inhibition performance have been investigated and presented in Fig. 2b,d, respectively.

According to Fig. 2(a), CMC samples with different degrees of carboxyl substitution showed different inhibition performance. Generally, the inhibition efficiency of CMC increased with the increase of carboxyl contents. As the degree of carboxyl substitution rose from 0.20 to 1.90, the optimal dose of CMC decreased and the corresponding inhibition efficiency increased (Table 1 and Fig. 2b), which was fully consistent with previous studies^24,31–33^. CMC(6) with the highest carboxyl contents in these six CMC samples showed the maximum efficiency approximately 98.3% at its optimal dose of 20.0 mg L^−1^, nearly complete inhibition of CaCO_3_ scale formation. However, CMC(1), CMC(2), and CMC(3) exhibited low inhibition efficiencies (< 80%) and high optimal doses (≥ 60.0 mg L^−1^) due to their relatively low carboxyl contents contained. CMCs can chelate with calcium ions and disperse scale-forming substances mainly via introduced carboxyl groups, which would decrease the scaling potential, thereby inhibit crystal growth and prevent formation of CaCO_3_ scale. Higher degree of carboxyl substitution usually resulted in enhanced chelation and complexation effects between CMC and calcium ions^24,33,52^. Moreover, increased carboxyl contents on CMC backbone made the spatial distance between the two adjacent carboxyl groups closer, which caused the Ca–CMC complex more easily formed and stable due to the synergistic effect, thereby enhancing the inhibition effects^53^.

### Effect of grafting ratio of CMC-*g-*PAA

On the basis of Fig. 2c, the effect of grafting ratio on the inhibition performance of CMC-*g*-PAA was summarized and shown in Table 2 and Fig. 2d. Differently from CMC (Fig. 2a), the inhibition performance of CMC-*g*-PAA roughly showed two stages with the increase of the grafting ratio. CMC-*g*-PAA(1)–CMC-*g*-PAA(4) with relatively low grafting ratios (24–241%) had higher inhibition efficiencies (> 90%) and lower optimal doses (< 10.0 mg L^−1^). Among them, CMC-*g*-PAA(2) showed a lower optimal dose approximately 6.0 mg L^−1^. With further increase of the grafting ratio, the required doses notably increased and the inhibition performance reduced, especially for CMC-*g*-PAA(6) and CMC-*g*-PAA(7). When the doses rose to 40.0 mg L^−1^, the inhibition efficiencies of CMC-*g*-PAA(6) and CMC-*g*-PAA(7) were just around 80%. Besides, some gel-like substances were found at the bottom of the flasks when CMC-*g*-PAA(6) or CMC-*g*-PAA(7) was dosed, due to the formation of insoluble Ca-CMC-*g*-PAA complexes. The weakened inhibition performance of CMC-*g*-PAA(6) and CMC-*g*-PAA(7) was ascribed to their too high grafting ratio^25,54,55^.

Generally, introduced PAA grafted chains would increase the content of carboxyl groups and improve the chelation and dispersion effects, thus enhancing the inhibition performance^25^. However, for these seven CMC-*g*-PAA samples (CMC-*g*-PAA(1)–CMC-*g*-PAA(7)) with the same grafted-chain number (N) (Table 2), the grafted-chain length (L) would increase with the increase of grafting ratio, because the grafting ratio was proportional to the product of L and N^27^. Higher grafting ratio here led to longer PAA grafted chains and increased molecular weight, which evidently enhanced the formation of intermolecular hydrogen bonds and the bridging flocculation effect of CMC-*g*-PAA^25,49,54–56^. The much higher grafting ratio thus caused the easy formation of insoluble Ca-CMC-*g*-PAA complexes and the notable reduction of the inhibition performance of CMC-*g*-PAA^25^. Therefore, the grafting ratio of CMC-*g*-PAA should be controlled at an appropriate range to achieve an effective inhibition performance.

### Effects of chain architecture on inhibiting CaCO_3_ scale formation: Distributions of carboxyl groups

Different from small-molecule antiscalants, distinctive chain architecture of polymeric antiscalants, i.e. linear, branching, star-like structures, would result in different distribution of functional groups, thus influencing the inhibition performance^41^. In addition, for graft copolymers, grafted-chain distribution including L and N is also a specific structural factor, which caused different distribution of grafted functional groups and notably affected the application properties^25^. Here, the effects of chain architecture were investigated and discussed in term of the detailed distributions of carboxyl groups on the polymeric backbone and branched chains.

CMC and CMC-*g*-PAA have far different chain architectures (Fig. 1), and the carboxyl groups randomly distributed on the backbone of linear CMC^43^ while the same functional groups were mainly situated on the branched chains of CMC-*g*-PAA^27^. The effects of chain architecture of these cellulose-based antiscalants on the inhibition performance were further investigated and compared. The two pairs of CMC and CMC-*g*-PAA, i.e. CMC(5) and CMC-*g*-PAA(2) and CMC(6) and CMC-*g*-PAA(3) had similar contents of carboxyl groups to each other; however, not only CMC-*g*-PAA(2) but also CMC-*g*-PAA(3) had notably lower optimal doses and better inhibition efficiencies based on Fig. 2 and Tables 1 and 2. Besides, CMC(6), the most efficient one in these six CMCs, contained more carboxyl groups but still showed lower inhibition performance than CMC-*g*-PAA(1) and CMC-*g*-PAA(2). CMC-*g*-PAAs thus exhibited better inhibition performance than CMCs. The obvious differences in their inhibition performance were originated in the different chain architectures between CMC and CMC-*g*-PAA.

In comparison to linear CMC, CMC-*g*-PAA exhibited multi-dimensional spatial structure in solutions due to its abundant branched chains (Fig. 1), which could facilitate to interact with scale-forming substances including cations and micro-crystals, thus greatly enhancing its chelation and dispersion effects, efficiently lowering the local *S* and reducing the scaling potentials, and notably improving the scale-inhibition performance^41,49^. This finding indicated that the branching architecture was a superior polymeric structure for those cellulose-based antiscalants containing carboxyl groups in improving their inhibition performance, besides, the chain architecture of polymeric antiscalants may even have more contributions than the content of carboxyl groups contained. It was because a superior chain architecture of polymeric antiscalants can give not only a full utilization of the introduced active inhibition sites but also a favorable spatial structure for notably enhancing the chelation and dispersion effects. Besides, given the competitions between dispersion and bridging flocculation effects existed in graft polymeric antiscalants, a suitable grafting ratio was the prerequisite to achieve the superior advantage of branching architecture in its scale inhibition.

### Grafted-chain distribution of CMC-*g*-PAA

As mentioned above, CMC-*g*-PAA showed better inhibition performance than CMC when they have the similar or even lower contents of carboxyl groups due to the superior advantage of branching architecture. However, grafting ratio is just a primary parameter, which cannot reflect the fine structural information of graft copolymers, such as the detailed grafted-chain distributions including grafted-chain number and length^27^. In fact, those fine structural factors also notably affected their application performance^25,27,38,39^, in addition to grafting ratio. Specifically, CMC-*g*-PAA(a)–CMC-*g*-PAA(d) with similar grafting ratios but different L and N (Fig. 1c,d) were prepared and their inhibition performance against CaCO_3_ scale were exhibited and compared in Fig. 2e and Table 2. CMC-*g*-PAAs with higher N but shorter L of PAA grafted chains presented a sharper slope at the initial dose stage (Fig. 2f) and showed better inhibition performance with lower optimal dose (Table 2), which was fully consistent with our previous study^25^. The high N of PAA grafted chains made the two adjacent PAA grafted chains bind to calcium ions more efficient due to the closer spatial distance, causing Ca-CMC-*g*-PAA complex more stable and the inhibition effect enhanced^25,53^. Furthermore, high N of PAA grafted chains meant more terminal groups existed in CMC-*g*-PAA, which was more active in the interactions with scale-forming substances^25,34^. On the contrary, CMC-*g*-PAA with low N of PAA grafted chains and correspondingly long L may not only contain less active terminal groups but also exhibit enhanced bridging flocculation effect, resulting in reduced chelation and dispersion effects and worsened inhibition performance^25,49^. Therefore, the grafted-chain distribution of graft polymeric antiscalants should be designed and fabricated properly for obtaining desired inhibition performance besides the grafting ratio.

### Characterization: SEM and XRD

**Figure 3:**
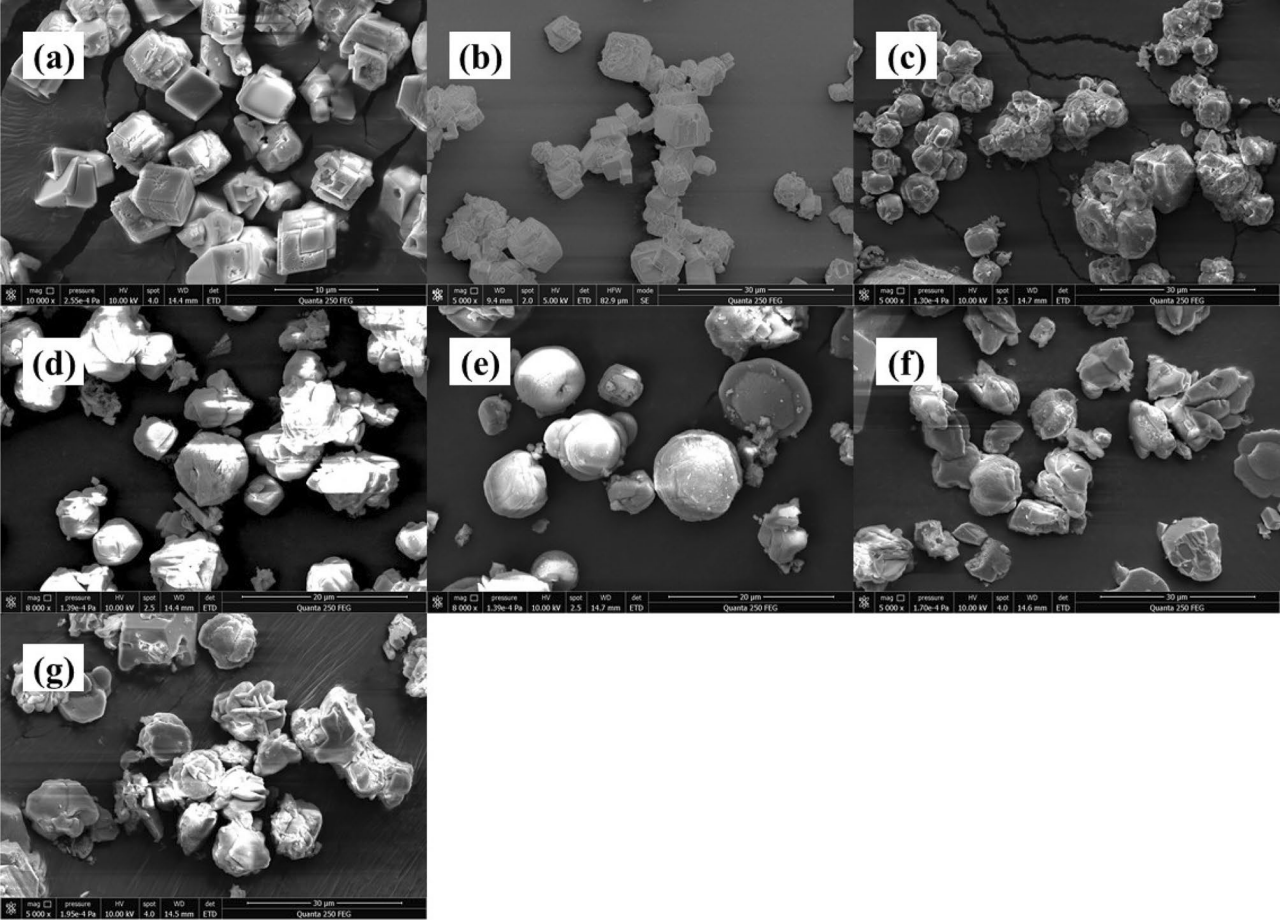
SEM images of CaCO_3_ prepared from CaCl_2_ and NaHCO_3_ aqueous mixtures (**a**) in the absence of antiscalants and (**b**–**g**) in the presence of various CMC samples with different degrees of carboxyl substitution: (**b**) CMC(1), (**c**) CMC(2), (**d**) CMC(3), (**e**) CMC(4), (**f**) CMC(5) and (**g**) CMC(6), respectively.

**Figure 4:**
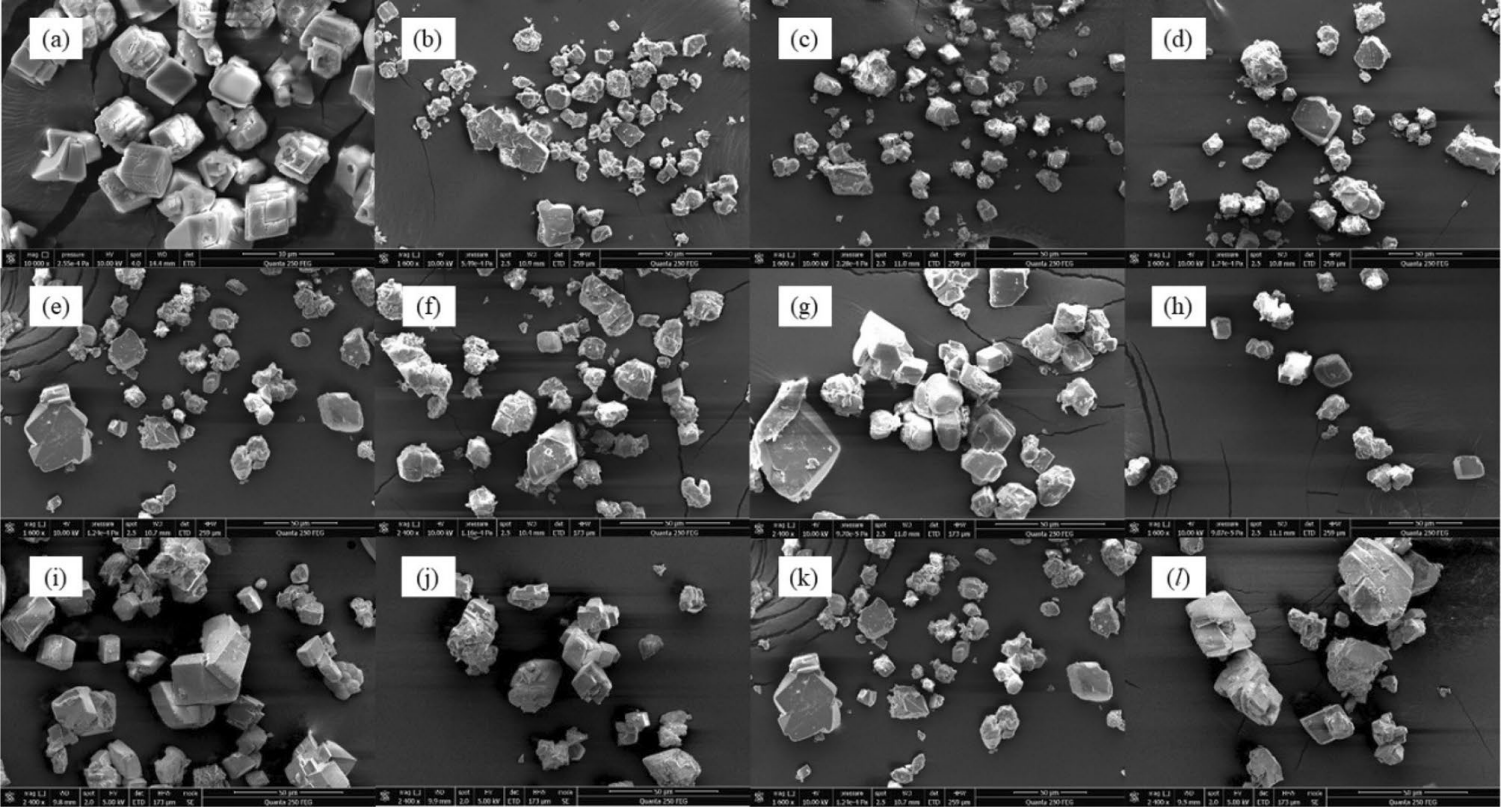
SEM images of CaCO_3_ prepared from CaCl_2_ and NaHCO_3_ aqueous mixtures (**a**) in the absence of antiscalants; (**b**–**h**) in the presence of various CMC-*g*-PAA samples with different grafting ratio: (**b**) CMC-*g*-PAA(1), (**c**) CMC-*g*-PAA(2), (**d**) CMC-*g*-PAA(3), (**e**) CMC-*g*-PAA(4), (**f**) CMC-*g*-PAA(5), (**g**) CMC-*g*-PAA(6) and (**h**) CMC-*g*-PAA(7); and (**i**–**l**) in the presence of various CMC-*g*-PAA samples with different grafted-chain distributions: (**i**) CMC-*g*-PAA(a), (**j**) CMC-*g*-PAA(b), (**k**) CMC-*g*-PAA(**c**) and (**l**) CMC-*g*-PAA(**d**), respectively.

**Figure 5:**
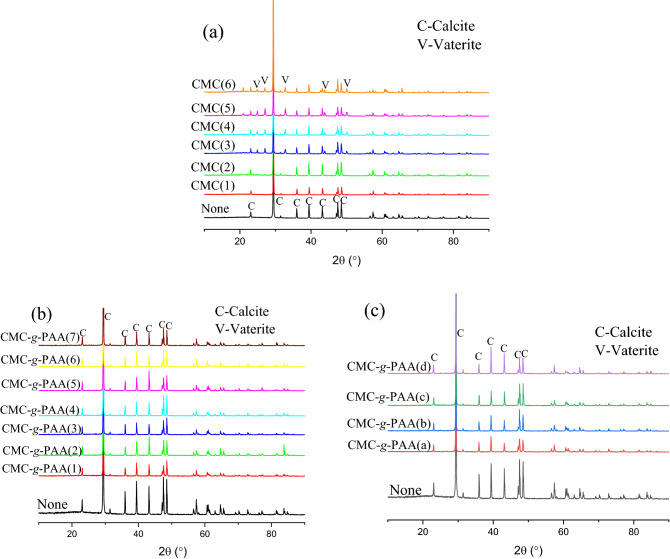
The XRD patterns of CaCO_3_ prepared from CaCl_2_ and NaHCO_3_ aqueous mixtures (**a**) in the presence of various CMC samples with different degrees of carboxyl substitution: CMC(1)–CMC(6), (**b**) in the presence of various CMC-*g*-PAA samples with different grafting ratios: CMC-*g*-PAA(1)–CMC-*g*-PAA(7), and (**c**) in the presence of various CMC-*g*-PAA samples with different grafted-chain distributions: CMC-*g*-PAA(a)–CMC-*g*-PAA(d). The black line in (**a**), (**b**) and (**c**) presents the XRD pattern of CaCO_3_ prepared from CaCl_2_ and NaHCO_3_ aqueous mixtures in the absence of antiscalants.

Scale deposits collected from static tests were further characterized by SEM and XRD to investigate the effects of antiscalants on CaCO_3_ crystal morphologies and the inhibition mechanisms (Figs. 3, 4, 5). Figure 3 presented SEM images of CaCO_3_ crystal deposits in the absence and in the presence of various CMC samples. The blank CaCO_3_ crystals had regular rhombohedrum shape, glossy surface (Fig. 3a), which showed a calcite structure. With the addition of CMC(1) and CMC(2), most formed crystals maintained a rhombohedrum shape, indicating their little crystal distortion effect. As CMC(3)–CMC(6) with the relatively high degrees of carboxyl substitution were fed, the obtained CaCO_3_ showed spherical/amorphous shapes and rough surfaces. The changes of CaCO_3_ crystal morphologies indicated that CMC may interrupt the normal growth of CaCO_3_ crystal and result in the formation of irregular crystals, which can be attributed to the adsorption of CMC on the active growth site of crystals. Moreover, more irregular crystals were observed in the presence of CMC with higher degree of carboxyl substitution, indicating that higher density of carboxyl groups can enhance the interactions between scale-forming substances and CMC, i.e., chelation with calcium ions and adsorption on crystals, thereby causing serious crystal distortion effect. This result was fully consistent with our previous study^33^.

XRD results of CaCO_3_ crystals formed in the presence of CMC were presented in Fig. 5a. The evident diffraction peaks at 23.08°, 29.40°, 35.96°, 39.40°, 43.16°, 47.48° and 48.52° in the blank CaCO_3_ samples indicated that calcite was the main form of the crystal samples^22,56^. The XRD patterns of crystals with addition of CMC(1) and CMC(2) were similar to that of blank CaCO_3_ crystal, while the different diffraction peaks at 24.88°, 27.08°, 32.76°, 43.84°, and 50.00° appeared in the XRD curves of crystal samples in the presence of CMC(3)–CMC(6), indicating the formation of the unstable CaCO_3_ crystal form, i.e., vaterite^22,56^. The XRD results were fully consistent with the above SEM observations, also confirming that CMC can inhibit the normal growth of crystals and cause crystal distortion by interacting with scale-forming substances and adsorption on the active growth site of crystals.

Figure 4 showed the SEM images of CaCO_3_ crystals obtained in the presence of various CMC-*g*-PAA samples (CMC-*g*-PAA(1)–CMC-*g*-PAA(7), Fig. 4b–h and CMC-*g*-PAA(a)–CMC-*g*-PAA(d), Fig. 4i–l). Differently from CMC (Fig. 3), the formed CaCO_3_ scales in the presence of various CMC-*g*-PAA samples mostly showed the same crystal configuration as that in the absence of antiscalant, except for some crystals exhibiting broken surface and edges; besides, the CaCO_3_ samples in the presence of various CMC-*g*-PAAs showed similar XRD patterns to that of blank CaCO_3_ samples (Fig. 5b,c). These findings indicated CMC-*g*-PAA may have some influence on the growth of CaCO_3_ crystal but had little distortion effects on final crystal structure. The introduced PAA branched chains led to increased content of carboxyl groups and drastically enhanced chelation and dispersion effects of CMC-*g*-PAAs, but the multi-dimensional spatial structure of CMC-*g*-PAA made it more difficult to enter the crystal lattices and interfere with CaCO_3_ crystal growth due to the steric hindrance^49^. However, linear CMCs are more easily accessible, thus showing obvious crystal distortion effect^57^. In short, SEM observation and XRD results both confirmed that CMC-*g*-PAA had little crystal distortion effect against CaCO_3_ scale but crystal distortion was one of the dominant scale-inhibition mechanisms for CMC.

### Effects of various structural factors on CaCO_3_ crystal growth

**Figure 6:**
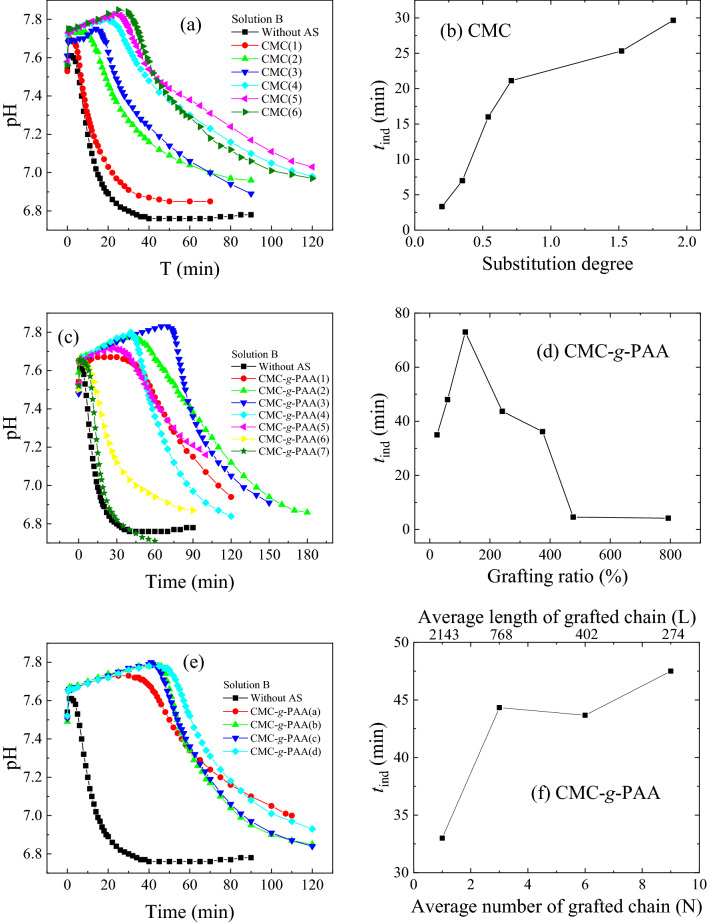
pH changes in the CaCO_3_ crystal growth process using Solution B in the absence of antiscalants and (**a**) in the presence of 2.0 mg L^−1^ CMC(1)–CMC(6), (**c**) in the presence of 1.0 mg L^−1^ CMC-*g*-PAA(1)–CMC-*g*-PAA(7), and (**e**) in the presence of 1.0 mg L^−1^ CMC-*g*-PAA(a)–CMC-*g*-PAA(d). According to (**a**), (**c**) and (**e**), the relations between *t*_ind_ and different structural factors, i.e., (**b**) degrees of carboxyl substitution, (**d**) grafting ratio, and (**f**) grafted-chain distribution, were summarized. (AS: antiscalant).

In order to further study the effects of those various cellulose-based antiscalants with different structures and their inhibition mechanisms, the CaCO_3_ crystal growth in the absence and in the presence of various antiscalants at a constant dose was detected by measuring the pH changes of calcium carbonate solution in the scaling process (Fig. 6a, c, and e). Accordingly, their *t*_ind_s were estimated and listed in Tables 1 and 2. After the addition of various CMC and CMC-*g*-PAA, the *t*_ind_s were all prolonged and crystal formation were delayed. This result was due to that CMC and CMC-*g*-PAA can inhibit crystal nucleation and delay crystal formation by interacting with calcium ions in solution and reducing local supersaturation ratio (*S*)^58^.

More interestingly, the effects of different structural factors on *t*_ind_ were fully consistent with those on their apparent inhibition efficiency (Fig. 2). Specifically, the *t*_ind_ increased with the increase of the degree of carboxyl substitution of CMC (Fig. 6b); CMC-*g*-PAA(1)–CMC-*g*-PAA(4) with relatively low grafting ratios (24–241%) had long *t*_ind_s, while CMC-*g*-PAA(6) and CMC-*g*-PAA(7) with higher grafting ratios had much shorter *t*_ind_s (Fig. 6d); among CMC-*g*-PAA(a)–CMC-*g*-PAA(d) with similar grafting ratios, CMC-*g*-PAA with more N but shorter L had relatively longer *t*_ind_ (Fig. 6f). Generally, increasing the content of carboxyl groups including the degree of carboxyl substitution of CMC and grafting ratio of CMC-*g*-PAA can enhance the chelation and dispersion effects, thereby effectively inhibiting crystal nucleation and crystal agglomeration^24,25,41,47,48^. However, higher grafting ratio would facilitate to form the intermolecular hydrogen bonds, enhance the bridging flocculation effect of CMC-*g*-PAA^25,49,54–56^, and cause the notable reduction of the inhibition performance^25^. CMC-*g*-PAA with higher N usually contained more active terminal groups in the interactions with scale-forming substances^25,34^ and thus exhibited higher inhibition efficiency. In short, those findings further confirmed that these structural factors of cellulose-based antiscalants played important roles in retarding CaCO_3_ crystal growth and inhibiting scale formation by various interactions with scale-forming substances.

### Effects of chain architecture on CaCO_3_ crystal growth

**Figure 7:**
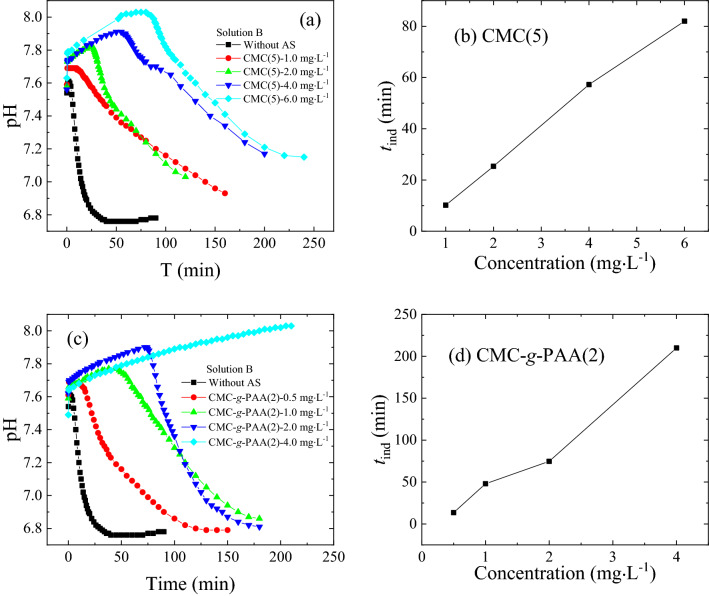
pH changes in the CaCO_3_ crystal growth process using Solution B in the absence of antiscalants and in the presence of different concentrations of (**a**) CMC(5) and (**c**) CMC-*g*-PAA(2). According to (**a**) and (**c**), the relations between *t*_ind_ and the different concentrations of various antiscalants, i.e., (**b**) CMC(5) and (**d**) CMC-*g*-PAA(2), were summarized.

**Figure 8:**
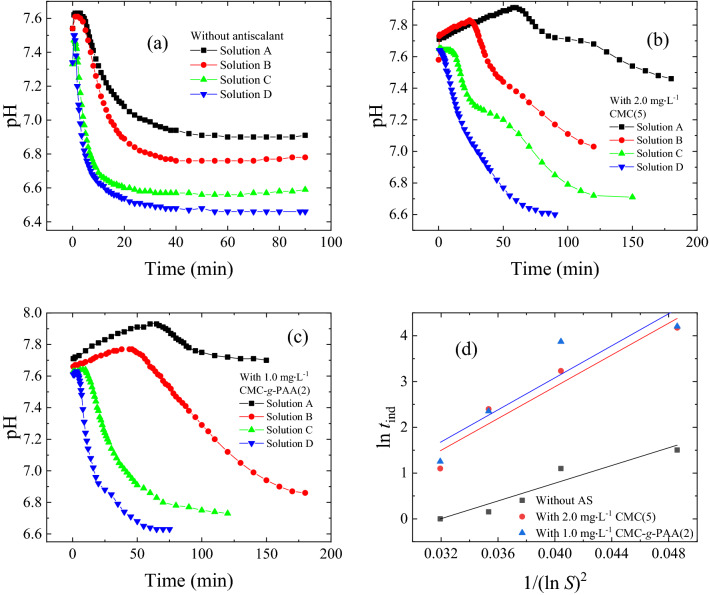
pH changes in the CaCO_3_ crystal growth process using four calcium carbonate solutions with various supersaturation ratios (**a**) in the absence of antiscalants, (**b**) in the presence of 2.0 mg L^−1^ CMC(5) and (**c**) in the presence of 1.0 mg L^−1^ CMC-*g*-PAA(2); (**d**) Relation between ln *t*_ind_ and 1/(ln *S*)^2^ without antiscalants, with 2.0 mg L^−1^ CMC(5) and with 1.0 mg L^−1^ CMC-*g*-PAA(2), respectively.

For further investigation of the effects of the chain architecture on these cellulose-based antiscalants, CMC(5) and CMC-*g*-PAA(2) were selected as the representatives of CMC and CMC-*g*-PAA due to their similar carboxyl contents. Figure 7a,c showed the pH changes of calcium carbonate solutions in the presence of various concentrations of CMC(5) and CMC-*g*-PAA(2), accordingly, their *t*_ind_s were summarized in Fig. 7b,d. *t*_ind_s increased with the increase of doses due to more carboxyl groups as inhibition sites introduced (Fig. 7a,c). At the same doses of 2.0 mg L^−1^, *t*_ind_ of CMC-*g*-PAA(2) about 70 min was much longer than that of CMC(5) around 25 min; moreover, *t*_ind_ of CMC(5) at doses of 6.0 mg L^−1^ was even much shorter than that of CMC-*g*-PAA(2) at doses of 4.0 mg L^−1^ (Fig. 7a,c). In addition, the surface energies of CaCO_3_ without antiscalant, with 2.0 mg L^−1^ of CMC(5), and with 1.0 mg L^−1^ of CMC-*g*-PAA(2) were calculated about 47.5, 57.7, and 57.8 mJ m^−2^, respectively, according to Eq. (2) by measuring the pH changes of various calcium carbonate solutions with different supersaturation ratios (Fig. 8). The obtained surface energies of CaCO_3_ were reasonable and were within the previously reported range of 31.0–62.0 mJ m^−2^^59–61^. The increased surface energies after dosing antiscalants indicated the inhibition effects of CMC and CMC-*g*-PAA on CaCO_3_ crystals growth may originate from increased surface energy and decreased nucleation rate of CaCO_3_ crystals^58,59,62^. The interactions between the cellulose-based antiscalants and scale-forming substances, including chelation, dispersion and adsorption on crystal surface, reduced the collision of scale-forming substances, decreased the solubility product of sparingly soluble salts and made crystal nucleation more difficult, effectively retarding the scaling processes. Besides, 1.0 mg L^−1^ of CMC-*g*-PAA(2) showed similar surface energies to 2.0 mg L^−1^ of CMC(5), indicating better inhibition effect of CMC-*g*-PAA(2). These results further confirmed the superior advantage of branching architecture of those cellulose-based antiscalants containing carboxyl groups against CaCO_3_ scale.

## Conclusion

In this work, two series of cellulose-based antiscalants with different molecular structures, i.e. CMC and CMC-*g*-PAA, were designed and prepared. In addition to dose effects, structural effects of these cellulose-based antiscalants on the inhibition of CaCO_3_ scale were investigated in detail via static tests and characterizations of CaCO_3_ crystal growth and final morphologies. The addition of CMC and CMC-*g*-PAA caused increased surface energy and *t*_ind_, decreased nucleation rates, and retarded CaCO_3_ scale formation. Besides, not only the contents of carboxyl groups but also the distributions of carboxyl groups played important roles in the inhibition performance of those cellulose-based antiscalants. CMCs with high degree of carboxyl substitution showed higher inhibition efficiency, lower optimal dose, and longer *t*_ind_. Suitable grafting ratio on CMC-*g*-PAA was favorable to avoid the formation of intermolecular hydrogen bonds and reduce bridging flocculation effect, and thus obtained better inhibition performance; with similar grafting ratios, more grafted-chain number led to increased inhibition efficiency of CMC-*g*-PAA and prolonged *t*_ind_ due to more active terminal groups contained. The distinct chain architectures of various cellulose-based antiscalants resulted in different inhibition mechanisms and scale-inhibition performance. The dominant scale-inhibition mechanisms of CMC-*g*-PAA should be the combination of chelation, dispersion and threshold effects; while crystal distortion was another important scale-inhibition effects for CMC. CMC-*g*-PAA with PAA branched chains exhibited much better inhibition performance than linear CMC, owing to notably enhanced chelation and dispersion effects originated in the distinct multi-dimensional spatial structure of graft copolymers in solutions. Although different graft polymeric antiscalants against various inorganic scales should be tried, these findings exemplify the importance of structure–activity relationship and offer some helpful guidance in the design and development of effective polymeric antiscalants.

## Experiment

### Reagents

Cellulose (particle size ˂ 25 μm) was purchased from Aladdin Industrial Co., Ltd. Monochloroacetic acid, sodium bicarbonate and sodium hydroxide were all purchased from Sinopharm Chemical Reagent Co., Ltd., and calcium chloride was from Xilong Scientific Co., Ltd. Acrylic acid (AA) was obtained from Tansoole Tech. Co., Ltd. and purified by vacuum distillation before use. Absolute ethyl alcohol (95%) was from Yasheng Chemical Co., Ltd. Ammonium persulfate, sodium borate, hydrochloric acid and acetone were purchased from Lingfeng Chemical Reagent Co., Ltd. All other chemicals were of analytical grade and used directly without further treatment. Distilled water was used in all experiments.

### Preparation and characterization

The two series of cellulose-based antiscalants, CMC and CMC-*g*-PAA, were obtained by etherification and graft copolymerization, respectively, as described in detail in Supplementary Information S1^24,27,43^. By adjusting the feeding masses of monochloroacetic acid (Table 1), CMC(1)–CMC(6) with different degrees of carboxyl substitution were obtained. Then, CMC-*g*-PAAs with various grafting ratios and grafted-chain distributions, namely CMC-*g*-PAA(1)–CMC-*g*-PAA(7) and CMC-*g*-PAA(a)–CMC-*g*-PAA(d), were prepared via graft copolymerization of AA monomers and CMC by controlling the feeding mass of ammonium persulfate and AA. CMC(3) was used in the synthesis of all CMC-*g*-PAA samples (Table 2).

A Fourier transform infrared spectrometer (FTIR, Bruker Model IFS 66/S) with wavelength range set between 500 and 4000 cm^−1^ and a ^1^H nuclear magnetic resonance (^1^H NMR) spectrometer (Bruker AVANCE model DRX-500) using D_2_O as the solvent at 500 MHz were used to characterize the molecular structure of the synthesized cellulose-based antiscalants. The degrees of carboxymethyl substitution in CMC samples were roughly estimated and obtained from the integral area of the characteristic peaks in their ^1^H NMR spectra^24^; while the grafting ratio of CMC-*g*-PAA was measured by the mass weight changes before and after graft copolymerization^27^; and the grafted-chain distribution of CMC-*g*-PAA was represented by the average number of grafted chain per graft copolymer (N) and the average number of the grafted monomers per grafted chain (L), which were roughly estimated on the basis of several assumptions^25,38,39^, as descripted in detail in Supplementary Information S1. An Ubbelohde-type capillary viscometer with 0.5–0.6 mm of diameter was employed to measure the intrinsic viscosity of each polymer solution based on one-point method^63^ (Supplementary Information S1). A Mettler Toledo pH meter was used for the measurement of pH in the scaling process. CaCO_3_ scales collected from static tests were observed by a FEI Quanta 250 FEG SEM under an acceleration voltage of 5 kV. The crystal forms of scale deposits were characterized by a powder X-ray diffractometer (Shimadzu model XRD-6000), operated at a voltage of 40 kV and current of 30 mA using Cu Ka radiation (λ = 0.15418 nm).

### Static test

According to the National Standard of China (GB/T16632-2008), the static scale-inhibition test against CaCO_3_ scale was conducted^64^. CaCl_2_ and NaHCO_3_ aqueous solution were thoroughly mixed, making final concentrations of Ca^2+^ and HCO_3_^−^ to be 5 mmol L^−1^. The antiscalant stock solution with the concentration of 1.0 g L^−1^ was freshly prepared. Different volumes of antiscalant stock solution were added to the above mixed solution for obtaining the sample solutions with different antiscalant concentrations ranged from 0 to 100 mg L^−1^ and the pH was adjusted to 8.0. The specimens were placed in a water bath at 70 °C for 10 h. After heating, the solution was filtered for the removal of the formed CaCO_3_ scale and cooled to the room temperature. 10 mL of the sample solution was taken out using a pipette to determine the concentration of residual Ca^2+^ by titration method recommended by the National Standard of China (GB/T16632-2008), which is widely used in scale-inhibition experiment^64^. The solution pH was adjusted to 12.0–13.0 with a dilute NaOH aqueous solution, and appropriate amounts of calcon-carboxylic acid indicator were added into the sample solution. The pre-prepared EDTA solution was used in the titration, the consumed volume of EDTA solution was recorded when the color of the solution changed from purple-red to bright blue, which was directly related to the residual Ca^2+^ in the filtrate^64^. A blank test without addition of antiscalants was conducted as control, thus the scale-inhibition efficiency of various antiscalants was calculated as follows,

1 $${\text{Inhibition efficiency (\% )}} = \frac{{V_{2} - V_{1} }}{{V_{0} - V_{1} }} \times 100\% ,$$

where *V*_0_ (mL) is the consumed volume of EDTA solution for titration of the specimen without addition of antiscalants and incubation treatment, while *V*_1_ and *V*_2_ (mL) are the consumed volumes of EDTA solution to the titration of the specimen in the absence and presence of antiscalants and heated for 10 h at 70 °C, respectively. Each test was all repeated at least 3 times and the final results were the average of three runs. The relative error of experimental results was below 5.0%.

### CaCO_3_ crystallization process

The effects of various cellulose-based antiscalants on the CaCO_3_ crystal growth were investigated for better understanding the scale-inhibition mechanisms. The crystallization process of CaCO_3_ was induced by mixing equal volume of CaCl_2_ and NaHCO_3_ solutions with equal molar concentration. The formation of CaCO_3_ scale would result in the reduction of pH due to the release of H^+^^16,65^. Thus, the *t*_ind_ of CaCO_3_ crystallization was measured by monitoring the change of pH. Four different concentrations of calcium carbonate solutions (0.015, 0.02, 0.025 and 0.03 mol L^−1^) were prepared and referred to as Solution A, B, C and D, respectively. The corresponding *S* was calculated using PHREEQC (version 3.4.0) and phreeqc.dat database, as shown in Supplementary Table S1. Appropriate amounts of various cellulose-based antiscalants were pre-mixed into sodium bicarbonate solutions for antiscalant-containing runs. Solution B was used in the investigation of dose and structural effects of various cellulose-based antiscalants. The pHs of CaCl_2_ and NaHCO_3_ solutions were both adjusted to 8.0 before mixing. Solution temperature was maintained at 25.0 ± 0.1 °C during the scaling process. The pH of the mixture under magnetic stirring was recorded at suitable time intervals. The *t*_ind_ can be regarded as the time point when the pH dropped obviously which was ≥ 0.01 in this study.

Based on the theory of classic homogeneous nucleation, the correlation between the *t*_ind_ and the *S* is as following^5,66^,

2 $$\ln t_{ind} = B + \frac{{\beta \gamma^{3} V_{m}^{2} N_{A} f\left( \theta \right)}}{{R^{3} T^{3} \left( {\ln S} \right)^{2} }},$$

where *B* is a constant; *β* is a geometric (shape) factor of 16π/3 for the spherical nucleus; *f*(*θ*) is a correction factor, which is equal to 1.0 for homogeneous nucleation and to < 1.0 for heterogeneous nucleation^58,66^; *V*_m_ is the molar volume of the phase forming (36.93 cm^3^ mol^−1^ for calcite); *γ* is the surface energy (J m^−2^); *N*_*A*_ is Avogadro’s number; *R* is the gas constant; and *T* is the absolute temperature.

## Supplementary information

Supplementary Information.
