# Supplementary material for: Chain architectures of various cellulose-based antiscalants on the inhibition of calcium carbonate scale
Source: Sci Rep. 2020 Dec 14;10:21906. doi: 10.1038/s41598-020-78408-w (PMC7736879; doi:10.1038/s41598-020-78408-w)
Supplement: Supplementary file 1 — Supplementary Information. [file 41598_2020_78408_MOESM1_ESM.docx]

**Supporting Information Cover Sheet**

**Chain architectures of various cellulose-based antiscalants on the inhibition of calcium carbonate scale ***

Wei Yu, Hu Yang**

State Key Laboratory of Pollution Control and Resource Reuse, School of the Environment, Nanjing University, Nanjing 210023, P. R. China

Number of pages: 12

Number of Table: 1

Number of Figure: 2

Number of Text: 1

* Supported by the National Natural Science Foundation of China (grant nos. 51778279 and 51978325).

** Corresponding author. Tel & Fax: 86-25-89681272, E-mail: yanghu@nju.edu.cn

**Table of Contents**

| **Index** | **Captions** | **Page** |
| --- | --- | --- |
| **Table S1** | Concentration, SI and *S* of the four different calcium carbonate solutions in CaCO_3_ crystallization process. | S3 |
| **Figure S1** | FTIR spectra of various cellulose-based antiscalants: (a) CMCs, (b) and (c) CMC-*g*-PAAs. | S4 |
| **Figure S2** | ^1^H NMR spectra of various cellulose-based antiscalants: (a) CMCs, (b) and (c) CMC-*g*-PAAs. | S5 |
| **Text S1** | Preparation and characterization of CMC and CMC-*g*-PAA. | S6–S11 |
| **References** |  | S11–S12 |

**Table S1** Concentration, SI and *S* of the four different calcium carbonate solutions in CaCO_3_ crystallization process.

| **No.** | **Calcium carbonate concentration (mol·L^-1^)** | **SI of calcite** | ***S* of calcite** |
| --- | --- | --- | --- |
| A | 0.015 | 1.97 | 93.325 |
| B | 0.020 | 2.16 | 144.544 |
| C | 0.025 | 2.31 | 204.174 |
| D | 0.030 | 2.43 | 269.153 |

^a^: Calcite was expected to be the main scale deposits, thus SI and *S* for calcite were presented here.

**Figure S1** FTIR spectra of various cellulose-based antiscalants: (a) CMCs, (b) and (c) CMC-*g*-PAAs.

**Figure S2** ^1^H NMR spectra of various cellulose-based antiscalants: (a) CMCs, (b) and (c) CMC-*g*-PAAs.

**Text S1** Preparation and characterization of CMC and CMC-*g*-PAA.

**Preparation of CMC**

CMC was synthesized on the basis of a reported method^S1^. First, 5.0 g of cellulose and 10 mL of 50% NaOH aqueous solution were added into a 70 mL of 95% ethanol solution and were incubated under mechanical stirring for 1 h in a water bath at 30 °C, such that the cellulose was fully swelled and alkalized. A certain amount of monochloroacetic acid was dissolved in a 20 mL of 95% ethanol solution, which was dropwise added into the reaction phase. After a 3 h reaction at 65 °C under total reflux, the mixture was adjusted to neutral pH using a dilute hydrochloric acid aqueous solution and then filtered to obtain a solid product. The obtained product was purified by filtration and rinsed to remove extra salt and unwanted byproducts using 85% ethanol and then vacuum dried in an oven at 60 °C for 48 h. Finally, the target product, namely, CMC, was successfully synthesized and stored at room temperature. Six CMC samples with different contents of carboxymethyl groups, which were named accordingly as CMC(1)–CMC(6), were obtained by adjusting the molar feeding ratio of monochloroacetic acid to cellulose.

**Preparation of CMC-*g*-PAA**

CMC-*g*-PAA was obtained via graft polymerization of CMC and AA monomers according to previous report^S2^. Abundant branched chains of PAA containing carboxyl groups were thus introduced onto CMC backbones. The same batch CMC(3) was used in the synthesis of all CMC-*g*-PAA samples. A desired amount of CMC was dispersed in deionized water with continuous stirring for 1 h at 70 ℃ under N_2_ atmosphere. After sufficient gelatinization, the CMC aqueous mixture was cooled down to 55 ℃ and a known amount of APS as the initiator was rapidly fed into the mixture. After 5 min, AA monomer solution was added to the CMC mixture dropwise. After a 3-h reaction under N_2_ atmosphere, the aqueous mixture was poured into acetone and the white precipitates were CMC-*g*-PAA. The crude product was washed and purified three times using ethanol, and dried in a vacuum oven at 60 ℃ for 48 h. Using acetone as the extractant, soxhlet extraction was conducted to further remove impurities. After full drying, the target product CMC-*g*-PAA was prepared. Various CMC-*g*-PAA samples with different structural morphologies were obtained by adjusting the feeding masses of the AA monomer and APS initiator.

**Viscosity measurement**

An Ubbelohde-type capillary viscometer with 0.5–0.6 mm of diameter was employed to measure the intrinsic viscosity of polymer solutions. The measured temperature was kept at 25.0±0.1 °C controlled by using a water bath. The antiscalant samples were diluted in 0.1 mol·L^-1^ NaCl aqueous solution. The efflux time of various antiscalant solutions in 0.1 mol·L^-1^ NaCl (t_u_) was measured, and that of solvent (t_v_, for 0.1 mol·L^-1^ NaCl) was also measured carefully. The relative viscosity of polymer solution (*η*_r_) was roughly equal to t_u_/t_v_. One-point method was used to estimate the intrinsic viscosity ([*η*])^S3^, which can indirectly reflect the molecular weight of antiscalant samples^S4^.

$[\eta\text{]=}\frac{\sqrt{2\times[\eta_{sp}-\ln(\eta_{r})]}}{C}$ (S1)

in which *C* (g·L^-1^) is the antiscalant concentration and *η*_sp_ is specific viscosity equal to (*η*_r_−1).

**Characterization of CMC**

The FTIR and ^1^H NMR spectra were measured to characterize the structures of CMC and CMC-*g*-PAA (Supporting Information Fig. S1 and S2). The new appeared characteristic peak at 1590 cm^−1^ in the FTIR spectra of all CMC samples was due to the introduced carboxymethyl groups compared to that of cellulose in Fig.S1(a)^S5^. And the new signals at 4.2–4.6 ppm in the ^1^H NMR spectra of CMC (Fig.S2(a)) were attributed to –CH_2_COO^-^ ^S6^. Analysis of FTIR and ^1^H NMR spectra both confirmed the successful preparation of CMC samples. Moreover, the peak area of the new appeared NMR signal becomes larger when adding more monochloroacetic acid in the synthesis process. The degrees of carboxymethyl substitution in CMC samples were roughly estimated and obtained from the integral area of the characteristic peaks in their ^1^H NMR spectra based on Eq.(S2), as listed in Table 1, which was accordingly increased from CMC(1) to CMC(6).

$\text{Degree of substitution}=\frac{Area(Hincarboxymethylgroup)/2}{Area(Hincellulosebackbone)/4}$ (S2)

**Characterization of CMC-*g*-PAA**

As shown in Figs. S1(b) and S1(c), the characteristic peaks of carboxyl groups of CMC-*g*-PAA samples have shifted to 1578–1550 cm^−1^, and new characteristic peak at 1454 cm^−1^ appeared in the FTIR spectra of CMC-*g*-PAA samples which can be attributed to –CH_2_– of the PAA grafted chains^S6^. From Figs. S2(b) and S2(c), the new signals at 1.17–1.67 ppm and 1.84–2.2 ppm in the ^1^H NMR spectra of CMC-*g*-PAA samples were attributed to –CH_2_– and –CH< in the PAA grafted chains, respectively^S7^. The spectra results indicated that CMC-*g*-PAA samples were successfully prepared.

The grafting ratio of CMC-*g*-PAA is the molar percentage of the AA monomer to a saccharide ring of CMC in CMC-*g*-PAA. The grafting ratio was measured by the mass weight changes before and after graft copolymerization, which was calculated as follows^S2^:

$\text{Grafting ratio}\left(\%\right)=\frac{W_{1}/72}{W_{0}/M_{\text{(CMC)}}}\times100\%$, (S3)

where *W*_0_ and *W*_1_ are the masses of the CMC backbone and the grafted PAA in CMC-*g*-PAA, respectively. The degree of carboxymethyl substitution of the selected CMC sample was estimated to be 0.54 on the basis of our previous result^S8^. The average molar mass of the saccharide ring of CMC, namely, *M*_(CMC)_, was thus calculated to be 193 according to Eq. (S4)

$M_{\text{(CMC)}}=M_{\text{(Saccharide ring)}}+M_{\text{(Carboxymethyl group)}}\times0.54=193$. (S4)

The grafted-chain distribution of CMC-*g*-PAA was represented by the average number of grafted chain per graft copolymer (N) and the average number of the grafted monomers per grafted chain (L). However, the actual N and L of a graft copolymer using current characterization methods is difficult to determine because of the uncertain initiation efficiency of initiator and graft copolymerization affected by various environmental factors^S9^. These two variables were approximately estimated using Eqs. (S5) and (S6) on the basis of several assumptions according to our previous work^S10-12^, which are also summarized in Table 2.

$\text{N}=\frac{2\cdot\eta\cdot m_{(\text{APS})}\cdot M_{(\text{CMC})}}{M_{(\text{APS})}\cdot m_{(\text{CMC})}}$, (S5)

$\text{L}=\frac{m_{(\text{AA})}/M_{(\text{AA})}}{m_{(\text{CMC})}/M_{(\text{CMC})}\times\text{N}}$, (S6)

where *m*_(APS)_, *m*_(AA)_, and *m*_(CMC)_ are the masses of APS, AA, and CMC, respectively. *M*_(APS)_, *M*_(AA)_, and *M*_(CMC)_ are the molar masses of APS, AA, and the saccharide ring of CMC, respectively. Meanwhile, *η* is the percentage of the actual disassociated APS, and this value is approximately 3.4% in this study when the reaction time is 3.0 h^S13^. In addition, the grafting ratio is proportional to the product of N and L^S2^, as shown in Eq. (S7).

$\text{Grafting ratio }\infty\text{ N}\times\text{L }$. (S7)

The grafting ratios of various CMC-*g*-PAAs were calculated according to mass weight change and the results are presented in Table 2. It can be seen that the grafting ratios increased from CMC-*g*-PAA(1) to CMC-*g*-PAA(7) with increase in the feeding mass of AA. With the same feeding masses of AA monomer, CMC-*g*-PAA(a)−CMC-*g*-PAA(d) showed similar grafting ratios. In addition, the N and L values of different CMC-*g*-PAAs were evaluated using theoretical estimation according to Eqs. (S5) and (S6), respectively (Table 2). Table 2 showed that L increased from CMC-*g*-PAA(1) to CMC-*g*-PAA(7), which was consistent with the change of the calculated grafting ratios. For CMC-*g*-PAA(a)−CMC-*g*-PAA(d), N theoretically increased with increase in the feeding mass of initiator but L decreased from CMC-*g*-PAA(a) to CMC-*g*-PAA(d), which was due to more grafting sites on CMC backbone from more initiated free radicals^S2^. The two series of CMC-*g*-PAA samples showed different structural morphologies, CMC-*g*-PAA(1)−CMC-*g*-PAA(7) had similar numbers of grafted chain but different grafting ratios, while CMC-*g*-PAA(a)−CMC-*g*-PAA(d) exhibited similar grafting ratios but different numbers of grafted chain.

**References**

S1. Wang, Y. W., Li, A. M. & Yang, H. Effects of substitution degree and molecular weight of carboxymethyl starch on its scale inhibition. *Desalination* **408**, 60-69 (2017).

S2. Susheel, K. & Sabaa, M. W. Polysaccharide based graft copolymers. Springer, Heidelberg, New York, Dordrecht, London (2013).

S3. Cheng, R. S. Extrapolation of viscosity data and calculation of intrinsic viscosity from one concentration of solution viscosity. *Polym. Bull. Chin.* **3**, 159-163 (1960).

S4. Williams, P. A. Handbook of Industrial Water Soluble Polymers. Blackwell Publishing Ltd., UK (2007).

S5. Cai, T., Li, H. J., Yang, R., Wang, Y. W., Li, R. H., Yang, H., Li, A. M. & Cheng, R. S. Efficient flocculation of an anionic dye from aqueous solutions using a cellulose-based flocculant. *Cellulose* **22**, 1439-1449 (2015).

S6. Ke, Y. K. & Dong, H. R. Analytical Chemistry Handbook. Third volume: Spectral analysis, second edition, Chemical Industry Press, Beijing (1998).

S7. Chen, Q., Yu, H. J., Wang, L., Abdin, Z., Yang, X. P., Wang, J. H., Zhou, W. D., Zhang, H. T. & Chen, X. Synthesis and characterization of amylose grafted poly(acrylic acid) and its application in ammonia adsorption. *Carbohydr. Polym.* **153**, 429-434 (2016).

S8. Yu, W., Song, D., Li, A. & Yang, H. Control of gypsum-dominated scaling in reverse osmosis system using carboxymethyl cellulose. *J. Membr. Sci.* **577**, 20-30 (2019).

S9. Fanta, G. F. Synthesis of graft and block copolymers of starch. In: Ceresa, R.J. (Ed.), Block and Graft Copolymerization, vol. 1. Wiley Inter. Science, New York, 1-27 (1973).

S10. Liu, Z. Z., Wei, H., Li, A. M. & Yang, H. Evaluation of structural effects on the flocculation performance of a co-graft starch-based flocculant. *Water Res.* **118**, 160-166 (2017).

S11. Yu, W., Wang, Y., Li, A. & Yang, H. Evaluation of the structural morphology of starch-*graft*-poly(acrylic acid) on its scale-inhibition efficiency. *Water Res.* **141**, 86-95 (2018).

S12. Hu, P., Xi, Z., Li, Y., Li, A. M. & Yang, H. Evaluation of the structural factors for the flocculation performance of a co-graft cationic starch-based flocculant. *Chemosphere* **240**, 124866 (2020).

S13. FMC Corporation. Persulfates technical information. http://www.peroxychem.com/media/90826/AOD_Brochure_Persulfate.pdf. (2001).
